# Early Detection and Long-Term Monitoring as a Strategy for African Swine Fever Outbreak Control and a Comparative Study on the Reproductive Performance of Convalescent and Naïve Sows in a Commercial Farm in Thailand

**DOI:** 10.3390/ani16081235

**Published:** 2026-04-17

**Authors:** Thanut Wathirunwong, Jatesada Jiwakanon, Klaus Depner, Sarthorn Porntrakulpipat

**Affiliations:** 1 Research Group for Animal Health Technology, Faculty of Veterinary Medicine, Khon Kaen University, Khon Kaen 40002, Thailand; thanut.w@kkumail.com (T.W.);; 2 Friedrich-Loeffler-Institut, 17493 Greifswald-Insel Riems, Germany

**Keywords:** African swine fever, convalescent pigs, early detection, reproductive performance, passive surveillance

## Abstract

African swine fever (ASF), caused by African swine fever virus (ASFV), is a highly destructive transboundary disease in domestic pigs. The circulating virus in this study belonged to ASFV genotype II, commonly associated with high virulence. In endemic regions such as Thailand, limited vaccine availability and shortages of naïve breeding stock necessitate reliance on early detection, surveillance, and the retention of convalescent sows, thereby raising concerns regarding viral persistence and reproductive performance. This study evaluated the long-term reproductive performance of convalescent sows compared with naïve cohorts under co-habitation conditions, while assessing the efficacy of passive surveillance and strict biosecurity in preventing viral transmission from both internal and external sources. Convalescent sows showed reproductive performance comparable to naïve cohorts across two parities. Long-term co-habitation with naïve sentinel pigs was not associated with detectable viral transmission, although low-level viral persistence or intermittent shedding cannot be excluded. From a disease control perspective, the transition from delayed detection to enhanced passive surveillance facilitated early clinical recognition and targeted removal (“tooth extraction”) of infected animals, effectively limiting intra-herd transmission without full depopulation. Importantly, irrespective of the uncertain carrier status, strict biosecurity and rapid response protocols appeared effective in mitigating both external introduction and within-farm transmission of ASFV. These findings suggest that, under appropriate management and biosecurity conditions, convalescent sows may be reintegrated into production systems with caution.

## Simple Summary

African swine fever (ASF) is a serious disease that causes major losses in pig farms worldwide. In countries like Thailand, where the disease is present, farms often face shortages of breeding animals after outbreaks. As a result, some farmers keep pigs that have recovered from infection, although there are concerns about whether these animals can still spread the virus or affect productivity. In this study, we followed a commercial pig farm after an outbreak of highly virulent ASFV genotype II and evaluated both disease control practices and reproductive performance. We found that pigs that had recovered from ASF produced litters at a similar level to naïve pigs. In addition, no horizontal virus transmission to naïve sentinel pigs was detected when recovered pigs were housed together over a long period. Importantly, improved farm management played a key role in controlling disease. Early recognition of clinical signs, combined with rapid removal of infected animals (“tooth extraction”), helped stop the spread of the virus without the need to cull the entire herd. Strict biosecurity and quick response measures were also effective in reducing the risk of both external introduction and within-farm transmission. These results suggest that, with careful management and strong biosecurity, recovered pigs may continue to be used in production systems. However, further research is needed to better understand the long-term risk of virus persistence.

## 1. Introduction

African swine fever (ASF) continues to represent one of the most severe transboundary animal diseases impacting the global swine industry, chiefly attributable to its near-100% case fatality rate and the lack of an effective vaccine or targeted treatment [1]. The etiological agent, African swine fever virus (ASFV), is a large double-stranded DNA virus from the Asfarviridae family, distinguished by its intricate genome and capacity to provoke hemorrhagic fever in pigs [1,2]. Depending on viral virulence, host immunity, and environmental factors, clinical manifestations can range from acute, highly lethal forms to subacute or chronic presentation [3]. These biological characteristics make ASF exceptionally difficult to control once introduced into a production system. The economic consequences of ASF outbreaks are profound and multifaceted. In addition to direct losses from mortality and mandatory culling, ASF imposes substantial indirect costs, including movement restrictions, prolonged farm downtime, repopulation expenses, and long-term disruptions to breeding programs [4]. Among porcine infectious diseases, ASF stands unparalleled in its ability to destabilize national and regional swine industries [5]. The absence of available commercial vaccines in Thailand or effective antiviral treatments against ASFV exacerbates these challenges, underscoring the critical need for robust disease management strategies [6,7,8]. Consequently, early detection, rapid response, and stringent long-term monitoring protocols are paramount for mitigating the spread and impact of ASF [1,9,10]. However, timely recognition of the disease in affected populations remains a significant challenge due to the varied clinical presentations and the complex epidemiology of the virus [11]. Experimental and field studies show challenges in distinguishing mild or nonspecific ASF signs from other hemorrhagic septicemic diseases, complicating early diagnosis [12,13]. Consequently, delayed disease suspicion often postpones sample submission and laboratory confirmation, allowing the virus to spread undetected within herds and across geographic regions [9]. This delay not only contributes to the rapid spread of the pathogen but also significantly amplifies the economic devastation caused by widespread culling and trade restrictions [4,14].

Molecular detection methods, particularly real-time polymerase chain reaction (PCR), have emerged as the gold standard for rapid, sensitive, and specific identification of the ASFV genome in clinical samples, thereby facilitating prompt diagnostic confirmation and enabling swift implementation of control measures [3,9,15]. Owing to these advantages, international authorities such as the Food and Agriculture Organization (FAO) and the World Organisation for Animal Health (WOAH) formally recommend PCR as the primary diagnostic method for ASFV detection in surveillance, control, and eradication programs [16]. The ability to rapidly confirm infection enables timely implementation of control measures, thereby limiting virus spread and reducing economic losses [13]. In numerous ASF-endemic countries worldwide, including Thailand in Southeast Asia, the sustained circulation of ASFV has precipitated acute shortages of breeding stock, exorbitant repopulation costs, and inadequate financial compensation for affected producers [4]. Confronted with these challenges, several farms have implemented the practical approach of retaining ASF-convalescent pigs to support herd reconstitution. Although financially justifiable, this strategy elicits critical epidemiological and production concerns, particularly pertaining to protracted viral persistence and the reproductive performance of recovered animals. A comprehensive understanding of these factors is crucial for evaluating the long-term viability and biosecurity implications of integrating convalescent animals into commercial breeding programs, especially given the virus’s ability to persist in the natural environment and infect through contact with contaminated environments [17]. Recent field data from Vietnam demonstrated that convalescent gilts could resume reproductive activity and farrow successfully without detectable persistent viremia, viral shedding, or vertical transmission over a 14-month period. However, reproductive performance was compromised compared with pre-outbreak records, including reduced litter size and increased reproductive failure [18]. Despite these findings, important gaps remain. Previous studies have primarily relied on comparisons with historical data rather than contemporaneous control groups, and information on long-term co-housing of convalescent and naïve animals under commercial conditions is limited. Furthermore, data on reproductive performance across successive parities following ASF recovery remain scarce. Concerns about viral persistence in pigs recovered from ASF add complexity to herd management choices. While most research indicates no strong evidence of ASFV transmission from such convalescent animals, transmission has occurred in certain scenarios, notably with pigs infected by moderately virulent strains like ASFV Netherlands’ 86 [19]. These cases represent a carrier state, marked by ongoing viremia and persistent virus shedding. However, pigs that have recuperated from acute ASF infection generally exhibit no detectable ASFV in peripheral blood, indicating the absence of active viremia and a markedly reduced transmission risk under typical farm conditions. Nevertheless, various studies have documented extended retention of ASFV DNA or antigens in tissues of convalescent pigs such as lymphoid organs, tonsils, lungs, and spleen well beyond clinical resolution [2,20,21]. These findings highlight the importance of ongoing surveillance for recovered animals, particularly in field environments where stressors like pregnancy, lactation, or co-infections could trigger viral reactivation or hinder timely detection.

In this context, the integration of early detection with systematic long-term surveillance of ASF-recovered pigs constitutes a pivotal strategy for outbreak containment and sustainable production in endemic areas. Nevertheless, empirical evidence from commercial farm settings on the efficacy of this approach remains scarce. Accordingly, the current study evaluated early detection and extended monitoring as a unified strategy for ASF outbreak management on a commercial swine farm in Thailand. In particular, it compared reproductive performance between ASF-convalescent and naïve sows, assessed immune responses and viral persistence over a prolonged period, and analyzed the epidemiological ramifications of retaining recovered animals in production systems. These results offer pragmatic insights for reconciling disease control with herd sustainability and contribute to the development of context-specific management strategies for ASF in endemic contexts.

## 2. Materials and Methods

### 2.1. Biosecurity Measures Implemented at the Study Farm

The investigation was conducted at a farrow-to-wean farm situated in Khon Kaen Province, northeastern Thailand, with a capacity for approximately 600 sows. The production facilities comprised three gestation barns (G), two farrowing barns (F), and one nursery-replacement barn (N)

**Figure 1:**
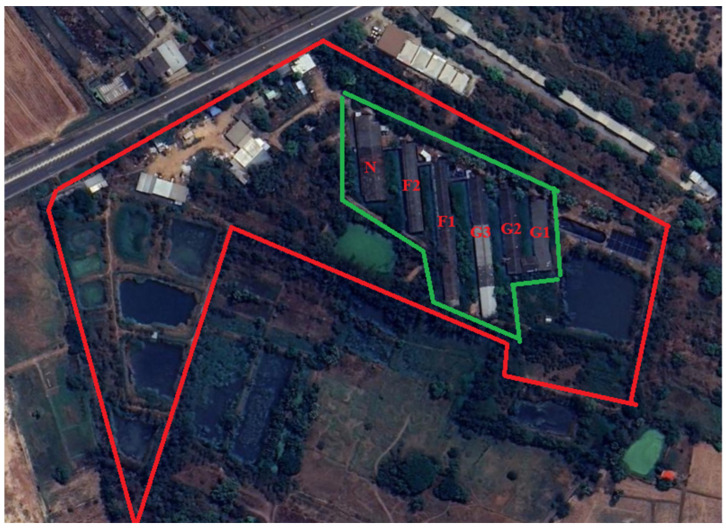
Biosecurity zoning of the study farm. The area enclosed within the red boundary represents the buffer zone, whereas the area within the green boundary represents the clean zone. Buildings are labeled as follows: G1–G3, gestation units; F1–F2, farrowing units; and N, nursery unit.

Following the initial 2021 ASF outbreak, which originated from a neighboring contract farm and progressed through the gestation and quarantine facilities despite aggressive containment efforts, the study farm underwent a transformative shift in its biosecurity infrastructure. Although the outbreak resulted in significant losses, the survival of 25 clinically healthy, ASFV DNA-negative animals, 17 of whom were seropositive, provided a critical window for recovery. After a rigorous three-month decontamination period confirmed by negative environmental PCR results, the facility implemented a comprehensive biosecurity framework modeled after Alarcón et al. (2021) to prevent pathogen reintroduction [22] (Figure 1).

**Figure 2:**
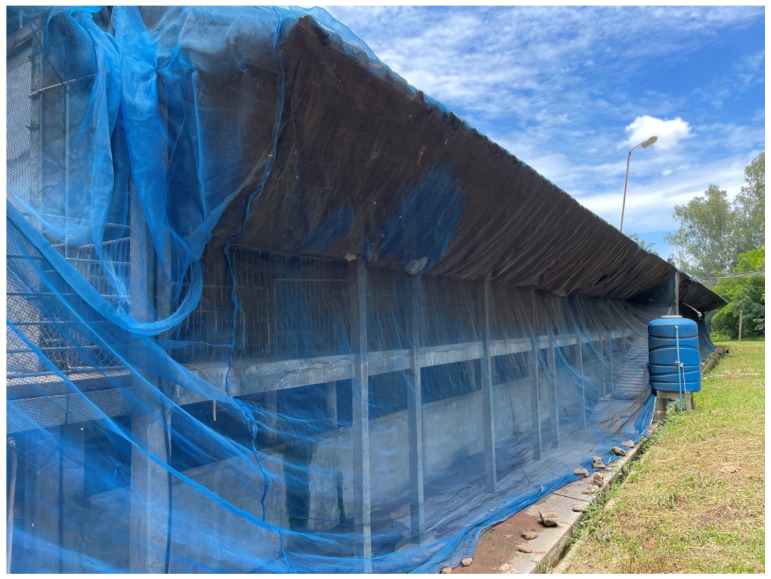
The implementation of insect-proof netting to reduce the risk of vector-borne transmission.

This enhanced protocol established a clear spatial hierarchy, categorizing the farm into clean, buffer, and dirty zones, each fortified by perimeter fencing to exclude external vectors such as stray animals (Figure 1). Movement between these zones was strictly regulated: personnel entering the buffer zone were required to use farm-specific attire, while entry into the clean production zone mandated a rigorous shower-in protocol and a secondary change in sterilized clothing. To mitigate vehicle-borne transmission, dedicated wash bays and footbaths were utilized at all access points. Furthermore, the internal production areas characterized by an open-house design were retrofitted with specialized netting to exclude insects and birds (Figure 2), thereby creating a multi-layered defense system aimed at long-term herd stabilization and biosafety.

Worker food consumption was categorically prohibited throughout the operational perimeter to preclude the ingress of contaminated pork products, a principal risk factor for ASFV introduction. During repopulation, the farm incorporated 144 replacement gilts and several boars, procured exclusively from an external ASF-free breeding nucleus. This approach adhered to international guidelines underscoring regulated animal sourcing and compulsory quarantine as cornerstone preventive measures. Subsequent herd augmentation proceeded internally via progeny of these foundational animals. The farm has since developed an in-house gilt rearing program and sustains an on-site boar station. The farm’s internal biosecurity practices, including personnel traffic management, barn-specific implements, and scheduled sanitation protocols, were engineered to constrain pathogen transmission within the premises in the event of agent incursion.

### 2.2. Disinfection Procedures and Environmental Sampling

Following total depopulation, a multistage decontamination protocol was implemented to eliminate ASFV from the farm environment. Initially, all potentially contaminated surfaces including pen floors, walkways, and equipment were treated with a sodium hydroxide solution (1:200 dilution) applied via watering cans to ensure uniform coverage. Facilities were then mechanically cleaned using detergent and brushes until free of visible organic matter, followed by a clean water rinse. A glutaraldehyde-based disinfectant (1:200 dilution) was subsequently applied to all surfaces. The farm underwent a structured fallow period consisting of an initial 14-day vacancy, followed by a second round of disinfection and an additional 14-day vacancy. During a final one-month period dedicated to structural repairs and biosecurity enhancement, a third round of disinfection was performed. Throughout the process, strict personnel biosecurity was enforced, including mandatory footwear disinfection with sodium hydroxide when exiting contaminated zones.

To validate the efficacy of the disinfection protocols, two rounds of environmental monitoring were conducted using real-time PCR. The first sampling occurred after the initial 14-day vacancy, while the second round was performed following the third disinfection phase to confirm a virus-free status prior to restocking. Environmental samples were collected using sterile gauze swabs moistened with 0.9% saline, targeting high-contact surfaces such as pen floors, feeders, and nipple drinkers. Additionally, soil and wastewater samples from the biogas system were collected to assess potential environmental persistence. All swabs were transported in sterile saline under refrigerated conditions (4 °C) for laboratory analysis. Repopulation was only initiated after all environmental samples tested negative for ASFV DNA, ensuring the absence of detectable environmental contamination.

### 2.3. Animals

All experimental procedures adhered to the Ethical Principles and Guidelines for the Use of Animals in Scientific Research promulgated by the National Research Council of Thailand and were approved by the Institutional Animal Care and Use Committee (IACUC) of Khon Kaen University (Protocol No. IACUC-KKU-90/67).

The sow line consisted of F1 Landrace × Yorkshire gilts derived from the farm’s long-established genetic lines, which historically produced ≤14 total born piglets per litter. Sows were categorized by parity into primiparous (P1; *n* = 20 litters) and second parity (P2; *n* = 20 litters) groups. In parity 1, all gilts were inseminated with semen from a convalescent Duroc boar, whereas in parity 2, sows were inseminated with semen from three naïve Duroc boars.

The study population comprised farm-origin pigs that survived the ASFV outbreak and newly introduced replacement animals from an external source. A total of 25 farm-origin survivors (24 females and 1 boar) were identified as clinically healthy and reached reproductive maturity at the time of screening. These survivors were categorized into two cohorts based on their immunological status: convalescent pigs (*n* = 17), defined as animals that survived the clinical outbreak and were confirmed seropositive by ELISA but PCR-negative for ASFV DNA; and naïve sows (*n* = 8), defined as farm-origin animals with no prior clinical history and confirmed seronegative and PCR-negative at the time of screening. To support herd repopulation, an additional 144 hyperprolific replacement gilts and boars (DanBred^®^, Vejle, Denmark) were introduced from a certified ASF-free external source. All introduced animals were confirmed to be both seronegative and PCR-negative upon entry and were managed under the same stringent biosecurity and surveillance protocols as the farm-origin cohorts throughout the monitoring period.

### 2.4. Animal Housing, Feeding, and Management

The production facilities comprised three gestation barns (G1–G3), two farrowing barns (F1–F2), and one nursery-replacement unit, organized according to production stage under standard commercial management conditions. The gestation barns included G1 and G2, each containing 150 individual sow crates, and G3 containing 220 crates. The farrowing facilities consisted of two barns, with F1 housing 96 farrowing crates and F2 housing 56 crates. The nursery-replacement unit comprised 126 pens distributed across 11 rooms, with 12 pens per room, designed to accommodate pigs from the post-weaning stage through to their development as replacement gilts. All units were managed under routine commercial production conditions with separation by production stage.

Sows were housed in an open system featuring mechanical ventilation fans and overhead water sprinklers to mitigate diurnal heat stress. These cooling measures are routinely employed in tropical swine production settings to attenuate the deleterious impacts of elevated ambient temperatures on sow welfare and reproductive efficiency [23,24]. The study was conducted under typical tropical conditions in northeastern Thailand, with ambient temperatures ranging from 24 to 34 °C and relative humidity between 65% and 85%.

During gestation, each sow was housed in an individual crate (0.65 × 2.0 m; 1.3 m^2^). Physical barriers between adjacent crates consisted of metal bars, allowing direct nose-to-nose contact between animals. Feeders were shared between adjacent gestation crates, whereas in the farrowing barn, each pen was equipped with a separate feeder. Each crate was equipped with an individual drinking system. Manure was manually collected by farm workers, placed into bags, and transferred to a biogas system. Drinking water, treated with chlorine dioxide (2 ppm), was provided ad libitum. Sows were fed a gestation diet formulated according to recommendations (2900 kcal/kg metabolizable energy, 16.0% crude protein, and 0.8% lysine), primarily based on rice bran and soybean meal. Feeding was conducted once daily at 08:00 h (2.0–2.2 kg/day) during weeks 1–11 of gestation and increased to 4.0 kg/day (divided into two meals) during weeks 12–15.

One week prior to the expected farrowing date, sows were moved to the farrowing house and housed in individual crates (1.95 × 0.75 m) within pens (2.5 × 3.0 m). Pens were fully slatted, with a concrete base for sows and steel slats for piglets. A heated creep area was provided for piglets during the first week postpartum. Sows were transitioned to a lactation diet (3200 kcal/kg metabolizable energy, 18.0% crude protein, and 1.0% lysine). Feed allowance was adjusted from 2.0 kg/day prepartum to 0.5–1.0 kg/day on the day of farrowing and gradually increased postpartum to 6.0 kg/day within one week. During weeks 2–3 of lactation, sows were fed ad libitum. Water was provided ad libitum via nipple drinkers.

The lactation period lasted 21–24 days. Boars were housed separately from sows within the same facility and were fed the same diet as lactating sows (2.5–3.0 kg/day) with ad libitum access to water. Parturition was monitored by trained personnel with minimal intervention. Assistance was provided only in cases of dystocia, defined as a birth interval exceeding 60 min. Routine daily health monitoring was conducted by a veterinarian.

Postpartum management included administration of an anti-inflammatory drug and vitamin/mineral supplement (tolfenamic acid, 40 mg/mL, 2 mg/kg, Tolfédine^®^, Vétoquinol, Lure Cedex, France) for 2 consecutive days after farrowing. Additionally, an antibiotic (amoxicillin, 1.0 mL/20 kg IM, 150 mg/mL, AMOXOIL^®^, Laboratorios Syva S.A.U., León, Spain) was administered on days 1 and 3 postpartum, and a vitamin/mineral supplement (217.8 mg/sow IM, Fercobsang^®^, Vétoquinol S.A., Lure Cedex, France) was given on day 1. Furthermore, all sows were vaccinated according to routine farm protocols against foot-and-mouth disease, porcine parvovirus (Synparv MRL^®^, Syva Laboratorios, León, Spain), classical swine fever (CSF, PRO-VAC^®^, Komipharm International Co., Ltd., Siheung, Republic of Korea), Aujeszky’s disease (Novoyesky^®^, Syva Laboratorios, León, Spain), and porcine reproductive and respiratory syndrome (PRRS, Pyrsvac-183^®^, Syva Laboratorios, León, Spain).

### 2.5. The Reproductive Performance Study

Reproductive performance was assessed in 16 sows that had recovered from ASF alongside 8 naïve sows. All replacement gilts underwent artificial insemination using semen from the ASF-recovered boar during their first parity and from naïve replacement boars during their second parity. Upon estrus detection, artificial insemination was conducted with boar semen pre-evaluated for sperm motility and concentration before and after dilution. Each gilt received two daily doses of 80 mL semen, supplemented by an additional insemination 12 h later if standing heat persisted. Evaluated metrics included farrowing rate, total piglets born (TB), live-born piglets (BA), proportion of mummified fetuses (MF), and proportion of stillborn piglets (SB), consistent with standard swine reproductive epidemiology indicators.

### 2.6. Longitudinal ASF Surveillance of the Entire Herd

Longitudinal monitoring for ASF across the entire herd was implemented from January 2022 to November 2024 using real-time PCR for ASFV detection in combination with ELISA assays. Passive surveillance involved continuous evaluation of blood samples from pigs displaying clinical signs consistent with ASF, including anorexia, lethargy, and pyrexia. EDTA-blood samples were collected for ASFV detection by real-time PCR, while serum samples were used for serological analysis (ELISA). Blood samples were also collected from all pig mortalities, with spleen tissue submitted for ASF confirmation by real-time PCR. To enhance early detection, all stockpersons and farm workers underwent targeted training to recognize ASF-compatible clinical signs and were instructed to immediately report suspect cases to the attending veterinarian. Diagnostic testing was performed in collaboration with the Veterinary Diagnostic Laboratory, Faculty of Veterinary Medicine, Khon Kaen University, where samples were processed and analyzed on the day of receipt to expedite results.

### 2.7. Culling Procedure

Once ASF infection was confirmed, all infected pigs were culled without delay to limit further disease spread. Small pigs were transported in sealed containers, while larger pigs were restrained and relocated using cages to promote safe handling and avoid environmental contamination. Following pig removal, personnel applied a sodium hydroxide solution to all paths traversed by pigs and staff, including pen interiors. At the operation’s conclusion, workers sanitized their boots with sodium hydroxide prior to leaving the infected area, meticulously cleaned and disinfected equipment, and showered. Clothing and personal protective equipment underwent overnight immersion in disinfectants prior to laundering and conditional reuse. The next day, the same personnel returned to scrub affected pen floors with detergent, then applied a glutaraldehyde-based disinfectant at a 1:200 dilution. To mitigate personnel-borne transmission risks, infected-area workers were barred from entering other pig housing areas. Throughout the ensuing 14-day monitoring period, pig movements within the affected barn were banned, access was confined to a single designated worker to curb inter-unit cross-contamination, and a thorough farm-wide assessment ran concurrently to detect any ASF dissemination beyond the impacted group.

### 2.8. Nucleic Acid Extraction, Real-Time PCR and Conventional PCR for ASFV Genotyping

Viral nucleic acid was extracted from porcine EDTA blood, serum, or organ tissue samples, according to sample availability, using the Vivantis Nucleic Acid Extraction Kit (Vivantis Technologies, Selangor, Malaysia) following an optimized protocol. Briefly, 200 µL of the sample was lysed with 200 µL of Lysis Buffer, vortexed, and incubated at room temperature for 10 min. After adding 200 µL of absolute ethanol, the lysate was transferred to a spin column and centrifuged at 10,000× *g*. The column was washed sequentially with Wash Buffer 1 and Wash Buffer 2, followed by an additional centrifugation to remove residual ethanol. The eluted viral DNA was stored at −20 °C until further use.

A real-time PCR assay targeting the highly conserved p72 gene (*B646L*) was performed. The diagnostic assay utilized specific primers targeting a conserved region of the ASFV genome: the forward primer (5′-CCC-AGG-RGA-TAA-AAT-GAC-TG-3′) and the reverse primer (5′-CAC-TRG-TTC-CCT-CCA-CCG-ATA-3′) (adapted from Fernández-Pinero et al., 2013) [25]. The amplification reaction was performed in a total volume of 25 µL, containing the extracted DNA template, optimized concentrations of primers (0.4–0.9 µM), and SYBR Green qPCR Master Mix (Thermo Scientific^®^, Waltham, MA, USA). Thermal cycling conditions consisted of an initial denaturation at 95 °C for 10 min to activate the DNA polymerase, followed by 40 to 45 cycles of denaturation at 95 °C for 15 s and a combined annealing/extension step at 58–60 °C for 1 min. Following amplification, a melting curve analysis was performed to verify the specificity of the PCR products. Fluorescence signals were captured during the annealing/extension phase. Samples were considered positive based on the presence of a characteristic amplification curve, a cycle threshold (Ct) value typically below 38, and a specific melting temperature (Tm) peak.

Genomic DNA fragments from ASFV-positive samples were amplified using specific primers targeting the *B646L* (p72), *E183L* (p54), and *CP204L* (p30) genes (Table 1). PCRs were performed using 2X AmPelify™ PCR Master Mix (BiotechRabbit GmbH, Hennigsdorf, Germany) in a final volume of 25 µL. The thermal cycling profile consisted of an initial denaturation at 95 °C for 2 min, followed by 40 cycles of denaturation at 95 °C for 30 s, gene-specific annealing for 30 s, and extension at 72 °C for 30 s, with a final extension at 72 °C for 5 min. Specifically, the annealing temperatures were optimized at 55 °C for *B646L*, 58 °C for *E183L*, and 60 °C for *CP204L*. Following amplification, the resulting PCR amplicons were purified using the Vivantis Nucleic Acid Extraction Kit according to the manufacturer’s instructions. A known ASFV-positive DNA sample and nuclease-free water were included as positive and negative controls, respectively, to ensure assay reliability.

### 2.9. ASF Antibody Detection Using Blocking ELISA Kit

Antibodies against ASF were detected in porcine serum samples employing the INGEZIM PPA COMPAC blocking ELISA kit. Serum samples were diluted 1:2 in the supplied diluent, with 50 µL of each added to wells of an antigen-coated microplate. Following a 1-h incubation at room temperature, unbound antibodies were removed via washing. Subsequently, 100 µL of peroxidase-conjugated anti-porcine immunoglobulin was introduced, followed by a 30-min incubation. Post-washing, 100 µL of substrate solution was added and incubated in the dark for 10 min. The reaction was halted with 50 µL of stop solution, and absorbance was measured at 450 nm.

ASFV-specific antibodies were determined by calculating the percentage inhibition per the manufacturer’s protocol. Samples with ≥50% inhibition were classified as positive, <40% as negative, and 40–50% as equivocal.

### 2.10. Sequencing and Phylogenetic Analysis of ASFV p30, p54, and p72 Genes

Purified PCR amplicons from conventional PCR were submitted to a commercial sequencing service for bidirectional Sanger sequencing (BIONICS^®^, Seoul, Republic of Korea). Sequencing reactions were performed using the BigDye™ Terminator v3.1 Cycle Sequencing Kit (Applied Biosystems, Foster City, CA, USA). Following a bead-based PCR product clean-up to remove unincorporated dye terminators, the samples were analyzed on an Applied Biosystems 3730XL DNA Analyzer (Applied Biosystems, Foster City, CA, USA). The resulting forward- and reverse-complement reads were manually inspected for quality, and consensus sequences were assembled for each gene fragment. These sequences were subsequently used for phylogenetic analysis and genomic characterization of the ASFV strains.

Phylogenetic analysis was performed based on the partial *B646L* (p72) gene, which is the standard marker for ASFV genotyping. The obtained sequences were compared with selected reference strains deposited in GenBank (Table 2). Sequence alignment was conducted using the CLUSTALW algorithm, and phylogenetic relationships were inferred using the maximum likelihood method with 1000 bootstrap replicates implemented in MEGA version 11 (MEGA11; Molecular Evolutionary Genetics Analysis, Pennsylvania State University, University Park, PA, USA) [29].

### 2.11. Statistical Analysis

All statistical analyses were performed using SAS version 9.4 (SAS Institute Inc., Cary, NC, USA). Descriptive statistics, including means, standard deviations, and minimum–maximum ranges for continuous variables, were generated using the MEANS procedure. Comparisons of reproductive performance parameters between ASF-convalescent and naïve sows were performed using Student’s *t*-test with Satterthwaite approximation for continuous variables. Normality of data distribution was assessed prior to analysis. Categorical variables, including conception rate and farrowing rate, were analyzed using Fisher’s exact test due to the small sample size. A *p*-value < 0.05 was considered statistically significant.

## 3. Results

### 3.1. Reproductive Performance

Reproductive performance was assessed for 16 convalescent sows and 8 naïve sows over two parities. No statistically significant differences were detected between convalescent and naïve sows across evaluated parameters, including conception rate, farrowing rate, gestation length, litter size, numbers of live-born and stillborn piglets, mummified fetuses, total weaned piglets, and culling rate in either type of parity. Thus, overall reproductive performance was comparable between groups (Table 3).

### 3.2. Disease Monitoring

**Figure 3:**
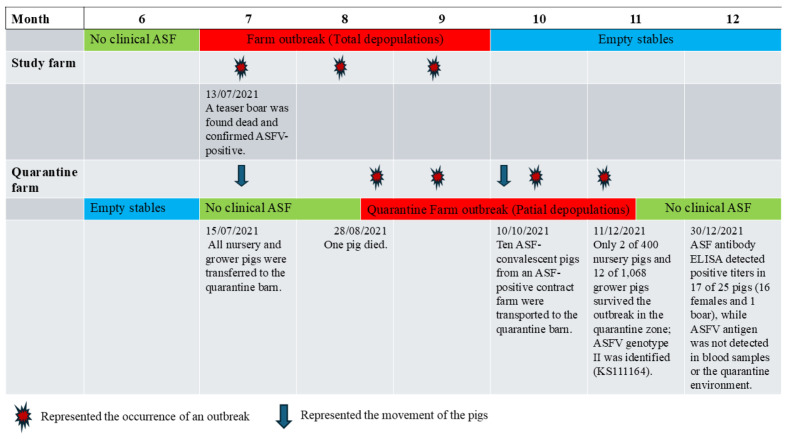
Disease monitoring during the outbreak (June–December 2021). Timeline of the ASF outbreak and pig movements at the study farm and quarantine farm following the initial identification of ASF at the contract farm on 1 June 2021 (KS240664).

On 1 June 2021, an ASF outbreak occurred at a wean-to-finish contract farm located approximately 20 km from the study farm, which served as the source of weanling pigs. ASFV infection at the contract farm was confirmed by PCR, and all pigs were subsequently culled. Approximately one month later, at the study farm, a teaser boar in the gestation barn (G2) was found dead without prior clinical signs. ASFV infection was confirmed by real-time PCR at an external laboratory one day later. In response, the farm owner immediately removed adjacent sows on both sides of the deceased boar and suspended all intra-farm animal movements. However, clinical cases soon emerged in pigs from non-adjacent sections, indicating rapid within-farm dissemination. Over the following week, the infection spread extensively across multiple barns, including G2 and G3, and progressed in an uncontrolled manner. As the outbreak intensified, all nursery and grower pigs were transferred to an off-site quarantine facility in an attempt to limit further transmission. When the outbreak proved uncontainable, all remaining on-site pigs, except those already relocated to quarantine, were culled. The outbreak persisted until early October 2021 (Figure 3). Due to the rapid progression of the outbreak, all breeding sows were depopulated within a short period, and it was not possible to determine the exact number of affected pens or sections within the sow herd.

In the quarantine facility, nursery and grower pigs that initially showed no clinical signs were co-housed. During routine farm management, pigs suspected of illness were collected from multiple pens and grouped into designated sick pens. Consequently, the original source of infection could not be determined, and transmission pathways could not be accurately reconstructed. By the time veterinary investigation was initiated, infected pigs had already been identified across multiple pens.

On 28 August 2021, a pig in the quarantine facility died and was confirmed positive for ASFV by real-time PCR, representing the first detected case within the quarantine barn. Following the death of this index case, pen mates developed clinical signs, including anorexia and lethargy, and the infection rapidly disseminated throughout the facility. The mixing of pigs from different pens, together with the aggregation of suspected cases, likely facilitated uncontrolled transmission within the quarantine barn, ultimately leading to the culling of all clinically affected animals.

At the end of October 2021, ten growing pigs that had survived an ASF outbreak at a separate contract farm were introduced into the same quarantine facility. Although these animals appeared clinically healthy at the time of introduction, subsequent disease progression was observed. By late November 2021, only 2 of 400 nursery pigs and 22 of 1568 growing pigs remained clinically healthy, while the remainder developed clinical signs and were culled. In addition, one boar survived without exhibiting clinical signs.

In total, 25 animals (24 crossbred Landrace × Yorkshire females and 1 Duroc boar) remained clinically healthy following the outbreak and had reached reproductive maturity at the time of reintroduction to the farm. Blood samples from all survivors were tested for ASFV DNA by real-time PCR and for ASFV-specific antibodies using ELISA. None of the animals tested positive for ASFV DNA in blood samples. Based on serological and virological results, 16 females and 1 boar were classified as seropositive (convalescent), while 8 females were classified as naïve, defined as animals that were seronegative by ELISA and negative for ASFV DNA by PCR at the time of post-outbreak screening (Table 4). These naïve animals were subsequently co-housed with seropositive animals under the same farm conditions during the monitoring period.

Following outbreak control, the study farm implemented rigorous cleaning and disinfection protocols over a three-month period prior to repopulation. All barns and equipment were thoroughly sanitized, and environmental sampling conducted after disinfection showed no detectable ASFV DNA by real-time PCR, supporting the effectiveness of the decontamination procedures under the conditions of this study. Environmental samples were collected and processed as described in Section 2.2.

### 3.3. Passive Surveillance for ASF

On 3 January 2022, the 25 surviving pigs were reintroduced to the disinfected barns after environmental swabs tested negative for ASFV DNA. Approximately two months later, 144 crossbred naïve gilts were acquired from an ASF-free integrated farm; these were subjected to random PCR testing for ASFV, with all samples yielding negative results. The surviving pigs and naïve gilts were subsequently reared together as the farm expanded its sow herd to 600 animals over the subsequent years.

From January 2022 to November 2024, pigs exhibiting clinical signs suggestive of ASF such as inappetence, depression, or fever were sampled per the passive surveillance protocol and tested for ASF virus in blood. Strict culling protocols, as detailed in the Materials and Methods section, were applied solely following laboratory confirmation of ASFV positive cases.

**Figure 4:**
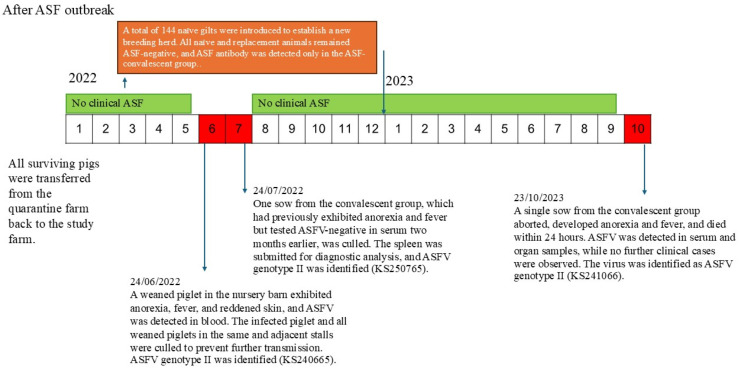
Timeline of ASFV-positive cases identified during the surveillance period.

As illustrated in Figure 4, three ASFV-positive cases were detected during the surveillance period. The first case, identified on 24 June 2022, involved a weaned piglet born to an ASF-recovered sow and housed in the nursery barn. The piglet exhibited reddened skin and reluctance to move. All pigs in the affected pen and those in adjacent pens were culled in accordance with the established containment protocol. No additional ASF cases were detected during the subsequent quarantine period.

The second case involved a convalescent sow in the farrowing barn that had displayed clinical signs of emaciation and intermittent anorexia since June 2022. Blood samples collected on 14 June and 8 July 2022 tested negative for ASFV by PCR. On 24 July 2022, the sow was culled, followed by postmortem examination. Gross pathological findings included pronounced splenomegaly and hepatomegaly, severe hemorrhagic lymphadenopathy, gray hepatization of both lungs, and abundant clear yellow ascitic fluid. ASFV DNA was detected in the spleen but not in the blood. Notably, despite postmortem confirmation of ASFV positivity, no culling of adjacent sows was performed. Per the established culling protocol, the affected pen was thoroughly cleaned and disinfected, with all barn pigs quarantined for 14 days prior to resuming normal operations. No additional infections occurred during quarantine, and clinically healthy pigs adjacent to the affected sow were monitored.

The third case was identified on 23 October 2023, involving a pregnant convalescent sow in the gestation barn that had displayed no prior abnormal clinical signs. The sow aborted and died the following day. Blood and spleen samples from the deceased animal underwent laboratory examination for ASF infection, with PCR analysis confirming ASFV presence in both tissues. Following confirmation of infection, movement restrictions were imposed on all pigs in the barn; notably, no culling of adjacent sows was performed. Blood samples from in-contact sows were subsequently collected on days 3, 7, and 14 post-incident; all tested negative for ASFV by PCR, and no ASFV DNA or antibodies were detected during the observation period.

### 3.4. ASF Antibody Monitoring

On 21 June 2023, 12 pigs consisting of 1 boar, 4 convalescent sows, and 7 offspring of varying ages underwent random selection for ASFV antibody monitoring via blocking ELISA. All adult convalescent pigs exhibited robust positive antibody responses, with % blocking values of 118.97–126.68%, signifying long-lasting ASFV-specific antibodies well after recovery. Five recently weaned piglets (3–4 weeks old) showed % blocking values of 125.4–129.26%, demonstrating effective maternal antibody transmission via colostrum. Two 8-week-old nursery pigs tested antibody-positive, albeit one with a reduced blocking value, indicating the onset of maternal antibody waning with age.

### 3.5. Genotyping and Phylogenetic Analysis Based on Partial CP204L (p30), E183L (p54) and B646L (p72) Genes

A total of six ASFV isolates collected between 2021 and 2023 were included in the study (Table 5). One isolate (KS240664) was obtained from a wean-to-finish farm that had previously received pigs from the study farm. The remaining five isolates were subsequently obtained from pigs at different production stages within the study farm, including nursery, farrowing, and gestation units. All isolates were characterized based on partial sequences of the *B646L* (p72), *E183L* (p54), and *CP204L* (p30) genes and were classified as ASFV genotype II.

### 3.6. Sequence Alignment and Phylogenetic Analysis of Partial B646L (p72) Genes

Analysis of the nucleotide sequences of the partial *B646L* (*p72*) gene demonstrated complete conservation among the six ASFV isolates, with 100% sequence identity observed across all samples. The obtained sequences were also identical to those of reference ASFV genotype II strains reported between 2018 and 2024.

**Figure 5:**
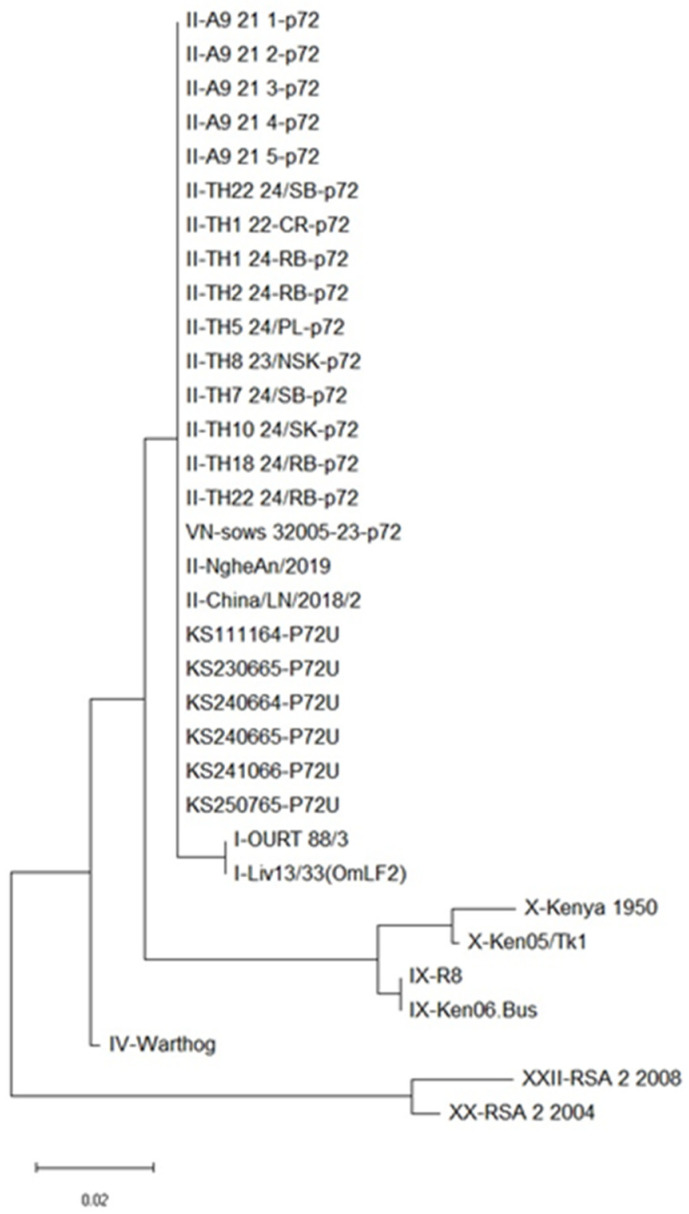
Phylogenetic relationships of ASFV inferred from nucleotide sequences of the p72 genes. Phylogenies were reconstructed using the maximum likelihood method implemented in MEGA11 software (https://www.megasoftware.net; accessed on 9 January 2026). Bootstrap support values greater than 50% (based on 1000 replicates) are shown at the corresponding nodes.

Phylogenetic analysis based on the partial *B646L* (*p72*) gene, using alignment with representative reference strains, consistently clustered all six isolates within ASFV genotype II, with no observable genetic divergence (Figure 5).

Although additional genomic regions (CP204L [p30] and E183L [p54]) were also analyzed, these sequences exhibited identical results, showing 100% identity among the study isolates and complete concordance with genotype II reference strains.

Accordingly, the viruses identified in this study were consistent with ASFV genotype II strains commonly associated with high virulence. This classification is further supported by the observed outbreak dynamics, in which most infected pigs developed acute disease and only 1.27% survived the infection.

## 4. Discussion

In the present study, conducted under commercial farming conditions, the reproductive performance of sows that recovered from ASFV infection proved comparable to that of naïve controls across two consecutive parities. Statistical analyses revealed no significant differences in key reproductive metrics, including litter size, farrowing rates, and total weaned piglets. These outcomes consistently aligned with the farm’s pre-outbreak historical benchmarks, demonstrating sustained productivity following exposure to a highly virulent genotype II strain. These results indicate that sows exhibiting clinical stability post-ASFV infection can restore normal reproductive performance, contrasting with earlier studies [18,30] that reported diminished productivity in such animals.

This clinical stability led to the classification of these animals as “type 2 survivors,” characterized by detectable antibodies coupled with an absence of viremia even upon re-exposure [31]. Consistent with observations by Petrov et al. (2018) [21], no viral transmission to other survivors or co-mingled naïve sentinel pigs was detected over a period exceeding 220 days post-infection. The restoration of physiological normalcy extended to the entire breeding cohort; notably, semen from a convalescent boar used during the initial parity showed no evidence of transmission to recipient sows or their offspring. This is consistent with prior reports indicating limited or absent transmission from ASFV convalescent pigs under natural conditions which suggests a reduced transmission risk under the conditions of this study [19,31].

The long-term carrier status of these convalescent sows requires careful interpretation as the findings of this study do not allow for a definitive classification of these animals as either “non-carriers” or “persistent carriers”. The continued presence of viral genetic material in tissues and high ELISA inhibition levels underscores a potential risk for recurrent viremia under stress; this, in turn, necessitates the implementation of rigorous passive surveillance strategies [32].

Regardless of the exact carrier status, the epidemiological evidence from this study suggests that viral shedding was either absent or remained below the threshold required for effective horizontal transmission under these conditions. The success of long-term herd management, therefore, depends heavily on the implementation of robust biocontainment and surveillance systems.

The catastrophic events during the June 2021 outbreak, inadequate biosecurity practices and a lack of passive surveillance facilitated the introduction of ASFV from external sources and delayed case identification. This allowed rapid viral dissemination across production units and into the quarantine barn before control measures could be implemented. The delayed detection and suboptimal response led to severe losses, necessitating total herd depopulation and a four-month production suspension for rigorous sanitation. By the time the infection was identified following the discovery of a deceased pig and multiple clinically affected animals in the quarantine barn, the virus had already propagated widely. Consequently, nearly the entire herd succumbed, yielding a survival rate of only 1.27% and underscoring how delayed interventions exponentially magnify the biological and economic impacts of ASF. In contrast, the incidents recorded in June 2022 and October 2023, following herd repopulation, highlight the critical efficacy of early identification and prompt containment. Although rigorous biosecurity protocols were in place, ASFV incursions still occurred, likely due to indirect transmission from external sources via biosecurity breaches, a common risk factor in commercial settings [33,34]. Crucially, vigilant observation of clinical signs by farm staff, paired with same-day laboratory confirmation, enabled immediate intervention. This rapid response is paramount; removing infected animals during the latent or early clinical phase prior to peak viral shedding significantly reduces the basic reproduction number (R0) and effectively halts intra-herd transmission chains [35]. Minimizing the time between infection and removal is essential to prevent the severe environmental contamination that typically occurs during the late acute phase [36]. Beyond curtailing viral shedding, strict biocontainment during the culling process is vital. Transporting infected animals in sealed, leak-proof containers and immediately disinfecting any secretions serve as critical barriers against residual environmental persistence [33,37]. Ultimately, the success of these containment efforts underscores the importance of the ‘human factor’ [33]. Continuous staff training to recognize early, non-specific signs (e.g., high fever, lethargy) is indispensable, as empowering stockmen to detect index cases before pathognomonic signs appear remains a key strategy for preventing large-scale outbreaks.

Notably, containment strategies evolved between the two later incidents. In June 2022, the protocol mandated culling the infected animal alongside all pen mates and pigs in adjacent pens deemed at risk. In October 2023, the response was highly targeted, with no culling of adjacent or contact pigs. This outcome provides empirical validation that rapid detection, coupled with immediate and appropriate intervention, can effectively halt intra-herd transmission even under minimized culling protocols [38].

Finally, several limitations of this study should be acknowledged. The irregular frequency of virological monitoring limits our ability to completely exclude intermittent or low-level viremia in convalescent pigs. Furthermore, ASFV detection relied solely on real-time PCR; without more sensitive approaches like virus isolation or in vivo bioassays, the absence of detectable viral DNA does not definitively confirm the absence of infectious virus. The lack of systematic post-mortem, histopathological, and tissue-based virological analyses restricts a comprehensive assessment of viral clearance. From a production perspective, reproductive performance evaluation was limited to farrowing outcomes and did not include broader metrics such as piglet weaning weight or growth performance. Lastly, the relatively small sample size and unequal group distribution may introduce selection bias, limiting the generalizability of the findings. These limitations underscore the need for future studies incorporating longitudinal sampling, advanced virological assays, and comprehensive production metrics to better elucidate the long-term implications of ASFV infection in convalescent pigs.

## 5. Conclusions

This study demonstrated that circulating viruses in the investigated farm belonged to the highly virulent ASFV genotype II. Convalescent sows, following recovery, showed reproductive performance comparable to naïve sows across two parities, suggesting that survivors may resume productivity under the specific conditions of this study. Furthermore, prolonged co-habitation of seropositive survivors with naïve sentinel pigs was not associated with detectable viral transmission during the observation period. However, due to limitations in virological surveillance and diagnostic sensitivity, the possibility of low-level viral persistence or intermittent shedding cannot be excluded.

From a disease control perspective, the findings highlight the potential importance of early detection and rapid intervention. While delayed detection initially resulted in substantial herd losses, the implementation of enhanced passive surveillance and early clinical recognition was associated with improved outbreak containment. Targeted removal (“tooth extraction”) may represent a practical approach under certain field conditions, although its effectiveness may depend on timely implementation and farm-specific factors. Importantly, the combined implementation of strict biosecurity measures and rapid response protocols appeared effective in mitigating both external introduction and within-farm transmission of ASFV, regardless of whether the potential source of infection originated from external incursions or persistently infected animals within the farm. Overall, while the findings provide valuable field-based insights, they should be interpreted with caution. Further studies incorporating longitudinal sampling, more sensitive detection methods, and comprehensive performance assessments are required to better understand viral persistence and transmission risk in convalescent pigs.

## Figures and Tables

**Table 1:** Oligonucleotide primers employed for ASF virus detection by PCR and for genomic characterization analyses.

Gene Name	Sequences (5′-3′)	Size of Products (bp)	References
*B646L*	GGCACAAGTTCGGACATGT	478	Bastos et al., 2003 [26]
	GTACTGTAACGCAGCACAG		
*E183L*	CGAAGTGCATGTAATAAACGTC	676	Gallardo et al., 2009 [27]
	TGTAATTTCATTGCGCCACAAC		
*CP204L*	ATGAAAATGGAGGTCATCTTCAAAAC	521	Rowlands et al., 2008 [28]
	AAGTTTAATGACCATGAGTCTTACC		

**Table 2:** Representative ASFV reference isolates selected for comparative analysis and used in the construction of the phylogenetic tree.

Isolates	Year of Isolation	Countries	Gene	Genotype	Genbank Accession Number
NgheAn/2019	2019	Vietnam	p72, p54, p30	II	MT180393
China/LN/2018/2	2018	China	p72, p54, p30	II	OR958825
VN/sows_32005/23	2023	Vietnam	p72, p54, p30	II	PQ329526
PL01	2022	Thailand	p72, p54, p30	II	OR567419
TH22_24/SB	September-2024	Thailand	p72, p54	II	PX126118, PX126126
TH18_24/RB	August-2024	Thailand	p72, p54	II	PX126117, PX126125
TH15_24/RB	August-2024	Thailand	p72, p54	II	PX126116, PX126124
TH10_24/SK	June-2024	Thailand	p72, p54	II	PX126115, PX126123
TH7_24/SB	January-2024	Thailand	p72, p54	II	PX126114, PX126122
TH5_24/PL	January-2024	Thailand	p72, p54	II	PX126113, PX126121
TH10_23/RN	October-2023	Thailand	p72, p54	II	PX126112, PX126120
TH8_23/NSK	July-2023	Thailand	p72, p54	II	PX126111, PX126119
A9_21_5	December-2022	Thailand	p72	II	OM461372
A9_21_4	December-2022	Thailand	p72	II	OM461371
A9_21_3	December-2022	Thailand	p72	II	OM461370
A9_21_2	December-2022	Thailand	p72	II	OM461369
A9_21_1	December-2022	Thailand	p72	II	OM461368
TH1_22/CR	October-2022	Thailand	p72, p54, p30	II	PP915735
TH1_24/RB	October-2024	Thailand	p72, p54, p30	II	PX119957
TH2_24/RB	October-2024	Thailand	p72, p54, p30	II	PX119974
OURT 88/3	1989	Portugal	p72, p54, p30	I	AM712240
Liv13/33 (OmLF2)	2017	France	p72, p54, p30	I	MN913970
Kenya 1950	1950	Kenya	p72, p54, p30	X	AY261360
Ken05/Tk1	2005	Kenya	p72, p54, p30	X	KM111294
Ken06.Bus	2006	Kenya	p72, p54, p30	IX	KM111295
R8	2015	Uganda	p72, p54, p30	IX	MH025916
Warthog	Not known	Namibia	p72, p54, p30	IV	AY261366
RSA_2_2008	2008	South Africa	p72, p54, p30	XXII	MN336500
RSA_2_2004	2004	South Africa	p72, p54, p30	XX	MN641877

**Table 3:** Comparison of reproductive performance parameters between ASF-Convalescent and Naïve sows across two parities.

Parameter	Parity 1		Parity 2	
	Convalescent (*n* = 16)	Naïve Sows (*n* = 8)	Convalescent (*n* = 16)	Naïve Sows (*n* = 8)
Mating (*n*/*N*)	16/16	8/8	16/16	8/8
Return to heat (*n*/*N*)	1/16	1/8	2/16	1/8
Conception rate (%)	93.75%	87.50%	87.50%	87.50%
Abortion (*n*/*N*)	1/16	1/8	1/16	0/8
Farrowing (*n*/*N*)	14/16	6/8	13/16	7/8
Gestation length (days)	115.8 ± 1.8	115.3 ± 1.3	116.4 ± 1.3	115 ± 1.4
Total number of piglets born per litter	11.4 ± 2.2	10.3 ± 3.2	11.1 ± 2.8	11.9 ± 2.8
Number of piglets born alive per litter	10.6 ± 2.3	9.67 ± 2.8	10 ± 2.6	11.6 ± 2.5
Stillborn piglets (%)	0.5 ± 0.7	0.67 ± 0.8	0.73 ± 0.9	0.22 ± 0.4
Mummified fetuses (%)	0.38 ± 0.6	0	0.4 ± 0.9	0.11 ± 0.3
Total weaned per litter	9.97 ± 1.9	9.67 ± 2.8	10 ± 2.2	11.56 ± 2.5
Culled sows (*n*/*N*)	0	0	1/16	0

No statistically significant differences were observed between convalescent and naïve sows for both continuous and categorical reproductive parameters across both parities (*p* > 0.05).

**Table 4:** ELISA-based detection of Anti-ASFV antibodies in pigs surviving an ASF outbreak.

Pig ID	Sex	% Blocking Antibody	Interpretation
1	Male	121.3	Positive
2	Female	24.0858	Negative
3	Female	20.9916	Negative
4	Female	98.6990	Positive
5	Female	99.6132	Positive
6	Female	98.1364	Positive
7	Female	96.3783	Positive
8	Female	98.2771	Positive
9	Female	23.5232	Negative
10	Female	19.0225	Negative
11	Female	117.7215	Positive
12	Female	116.7722	Positive
13	Female	120.2532	Positive
14	Female	110.7595	Positive
15	Female	104.4304	Positive
16	Female	88.1	Positive
17	Female	99.6	Positive
18	Female	122.9	Positive
19	Female	127.1	Positive
20	Female	126.6	Positive
21	Female	93.4	Positive
22	Female	10.3727	Negative
23	Female	16.98	Negative
24	Female	23.59	Negative
25	Female	20.14	Negative

**Table 5:** Summary of ASFV isolates used in this study.

Isolates	Year of Isolation	Status of Affected Pigs	Countries	Gene	Genotype
KS240664	2021	Nursery	Thailand	*p72*, *p54*, *p30*	II
KS111164	2021	Nursery	Thailand	*p72*, *p54*, *p30*	II
KS230665	2022	Nursery	Thailand	*p72*, *p54*, *p30*	II
KS240665	2022	Nursery	Thailand	*p72*, *p54*, *p30*	II
KS250765	2022	Farrowing	Thailand	*p72*, *p54*, *p30*	II
KS241066	2023	Pregnant	Thailand	*p72*, *p54*, *p30*	II

## Data Availability

The data that support the findings of this study are available from the corresponding author upon reasonable request.
